# Chronic pregabalin treatment protects against spreading depolarization and alters hippocampal synaptic characteristics in a model of familial hemiplegic migraine-type 1

**DOI:** 10.1186/s13041-023-01062-6

**Published:** 2023-11-03

**Authors:** Stuart M. Cain, Sascha R. A. Alles, Ray Gopaul, Louis-Philippe Bernier, Andrew C. Yung, Andrew Bauman, Yi Yang, Glen B. Baker, Piotr Kozlowski, Brian A. MacVicar, Terrance P. Snutch

**Affiliations:** 1 Michael Smith Laboratories, University of British Columbia, 219-2185 East Mall, Vancouver, BC V6T 1Z4 Canada; 2 Djavad Mowafaghian Center for Brain Health, University of British Columbia, Vancouver, Canada; 3 Department of Anesthesiology and Critical Care Medicine, University of New Mexico School of Medicine, Albuquerque, NM USA; 4 UBC MRI Research Facility, University of British Columbia, Vancouver, Canada; 5 Neurochemical Research Unit, Department of Psychiatry, University of Alberta, Edmonton, Canada

**Keywords:** Spreading depolarization, Pregabalin, Familial hemiplegic migraine, Spontaneous excitatory postsynaptic currents

## Abstract

Familial hemiplegic migraine type-1 (FHM-1) is a form of migraine with aura caused by mutations in the P/Q-type (Cav2.1) voltage-gated calcium channel. Pregabalin, used clinically in the treatment of chronic pain and epilepsy, inhibits P/Q-type calcium channel activity and recent studies suggest that it may have potential for the treatment of migraine. Spreading Depolarization (SD) is a neurophysiological phenomenon that can occur during migraine with aura by propagating a wave of silenced neuronal function through cortex and sometimes subcortical brain structures. Here, utilizing an optogenetic stimulation technique optimized to allow for non-invasive initiation of cortical SD, we demonstrate that chronic pregabalin administration [12 mg/kg/day (s.c.)] in vivo increased the threshold for cortical spreading depolarization in transgenic mice harboring the clinically-relevant Ca_v_2.1^S218L^ mutation (S218L). In addition, chronic pregabalin treatment limited subcortical propagation of recurrent spreading depolarization events to the striatum and hippocampus in both wild-type and S218L mice. To examine contributing underlying mechanisms of action of chronic pregabalin, we performed whole-cell patch-clamp electrophysiology in CA1 neurons in ex vivo brain slices from mice treated with chronic pregabalin vs vehicle. In WT mice, chronic pregabalin produced a decrease in spontaneous excitatory postsynaptic current (sEPSC) amplitude with no effect on frequency. In contrast, in S218L mice chronic pregabalin produced an increase in sEPSC amplitude and decreased frequency. These electrophysiological findings suggest that in FHM-1 mice chronic pregabalin acts through both pre- and post-synaptic mechanisms in CA1 hippocampal neurons to elicit FHM-1 genotype-specific inhibitory action. The results highlight the potential of chronic pregabalin to limit recurrent SD to subcortical brain structures during pathophysiological events in both the genetically-normal and FHM-1 brain. The work further provides insights into FHM-1 pathophysiology and the potential for chronic pregabalin treatment to prevent SD in migraineurs.

**Supplementary Information:**

The online version contains supplementary material available at 10.1186/s13041-023-01062-6.

## Introduction

Pregabalin is a widely prescribed first-line treatment for neuropathic pain conditions including post-herpetic neuralgia and diabetic neuropathy [1], and is also approved for the treatment of epilepsy [2]. Pregabalin belongs to a class of drugs called gabapentinoids shown to be high affinity ligands of the α2δ-1 and −2 ancillary subunits of High Voltage-Activated (HVA) calcium channels (for review see Alles et al. [1]). Pregabalin reduces HVA calcium channel activity to affect neurotransmitter release mechanistically acting directly and/or affecting calcium channel trafficking [3–7]. Gabapentinoids have also been shown to interact with thrombospondin receptors, BK potassium channels, NMDA receptors, α-neurexins, GABA-A receptors and prion proteins [1].

Migraine with or without aura affects up to 20% of the population with a higher prevalence in women than in men [8]. Migraine with aura is associated with a pathophysiological phenomenon known as spreading depolarization (SD), characterized by a sustained depolarization of neurons that propagates as a wave across the cortex at approximately 1–10 mm per minute [9–13]. Familial Hemiplegic Migraine Type 1 (FHM-1) is a monogenetic type of migraine with aura that in its most severe forms can manifest with accompanying muscle weakness and ataxia [8]. FHM-1 results from gain-of-function point mutations in the *CACNA1A* gene encoding the Ca_v_2.1 subunit of P/Q-type voltage-gated calcium channels [14, 15]. Transgenic mouse models harboring the clinically-relevant R192Q and S218L FHM-1 mutations in the *Cacna1a* channel gene reproduce several features of FHM-1, including increased susceptibility to cortical SD [14, 16]. The S218L mutation further results in a more clinically-severe phenotype, including ataxia and both non-fatal and fatal seizures [16, 17].

Clinical studies investigating the potential of pregabalin in the treatment of migraine attacks have demonstrated its effectiveness in both open-label and randomized controlled trials in children and in adults [18–21]. Our previous studies indicated that acute pregabalin administration in vivo (160 mg/kg, i.p.) both increased the threshold for cortical SD in wild-type mice and slowed cortical SD speed in R192Q and S218L transgenic mouse models [22]. Further, SD was demonstrated to propagate into the hippocampus and striatum in both FHM-1 strains (with a marked delay in the R192Q strain) with acute pregabalin treatment attenuating subcortical propagation of SD in the R192Q strain alone. However, a single acute pregabalin dose is not generally representative of clinical dosing where neuropathic pain and epilepsy patients are typically treated chronically at 150–600 mg per day for weeks to months [23]. Indeed, patients with neuropathic pain do not experience any pain relief until at least day 2 to 3 of pregabalin treatment [24] and in migraine patients pregabalin may not be effective until after several weeks of treatment [18, 21]. As such, it would be appropriate to evaluate pregabalin chronic treatment in animal models.

Here, utilizing an optogenetic stimulation technique optimized for non-invasive initiation of SD [25], we demonstrate that chronic pregabalin administration in vivo at a clinically relevant dose of 12 mg/kg/day (s.c.) increased the threshold for SD in FHM-1 mice harboring the more severe S218L mutation. In addition, chronic pregabalin treatment limited subcortical propagation of recurrent SD events to the striatum and hippocampus in both wild-type and S218L mice. Finally, we demonstrate that chronic pregabalin treatment had genotype-specific effects on synaptic excitability at hippocampal CA1 synapses in vitro, which elucidates a possible mechanism of action of chronic pregabalin in FHM-1.

## Results

### Chronic pregabalin treatment increases the threshold for cortical SD initiation in S218L FHM-1 mice

**Fig. 1:**
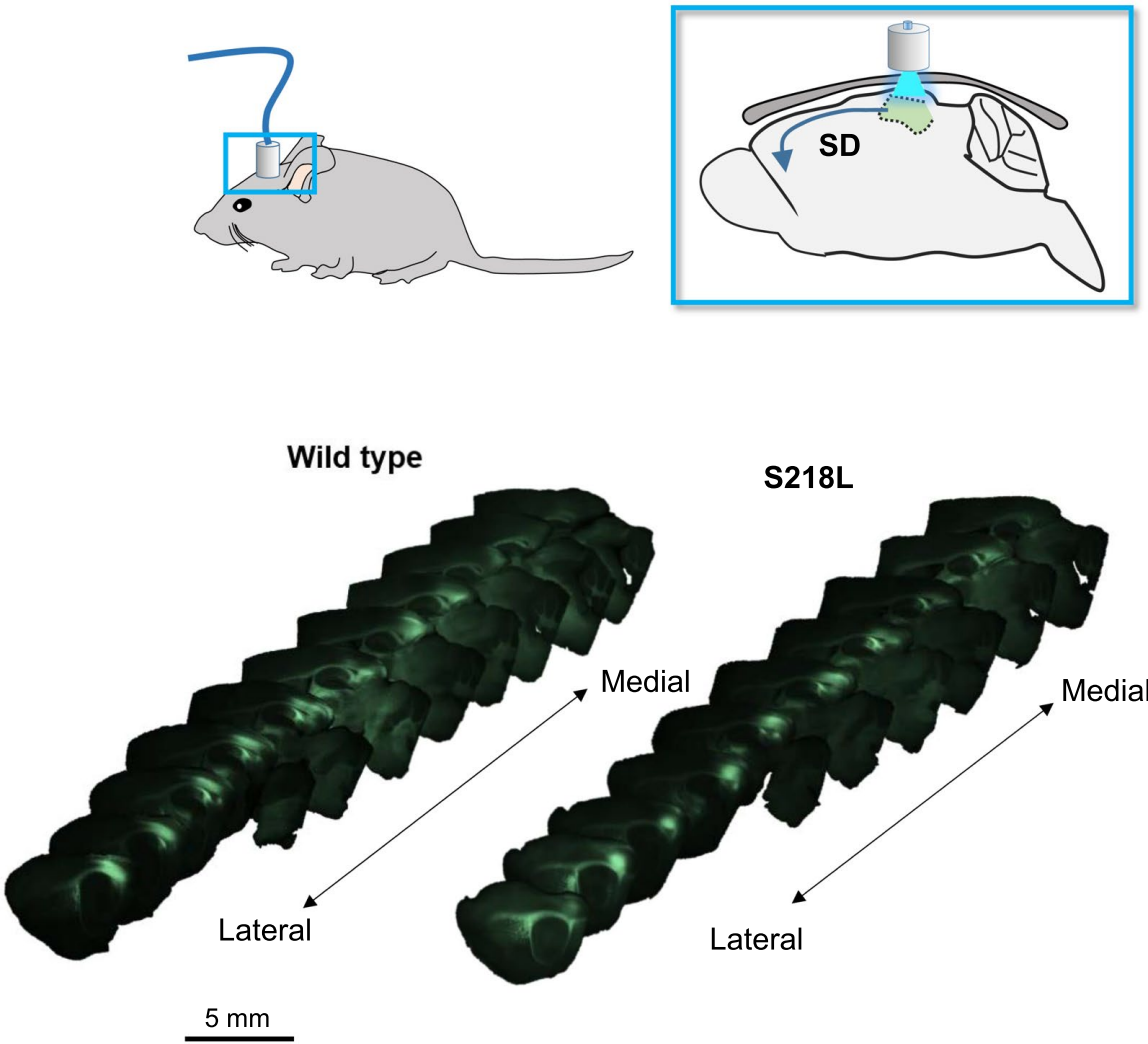
Experimental setup for DW-MRI experiments. (Upper panels) Schematic showing location of fiberoptic port on head and through-skull optogenetic stimulation relative to visual cortex in sagittal view. (Lower panels) Representative ex vivo immunofluorescence images of AAV5-hChR2(H134R)-YFP expression in sagittal plane, arranged from lateral to midline from animals used in DW-MRI studies

To evaluate the impact of chronic pregabalin treatment on SD in vivo, wild-type and S218L mice were chronically treated with pregabalin (12 mg/kg/day, s.c.) or vehicle for 7–9 days using an implanted osmotic minipump. SD was initiated by through-skull optogenetic simulation of the visual cortex, injected two-weeks previously with AAV5-hsyn-hChR2(H134R)-EYFP-AAV5 (Fig. 1).

**Fig. 2:**
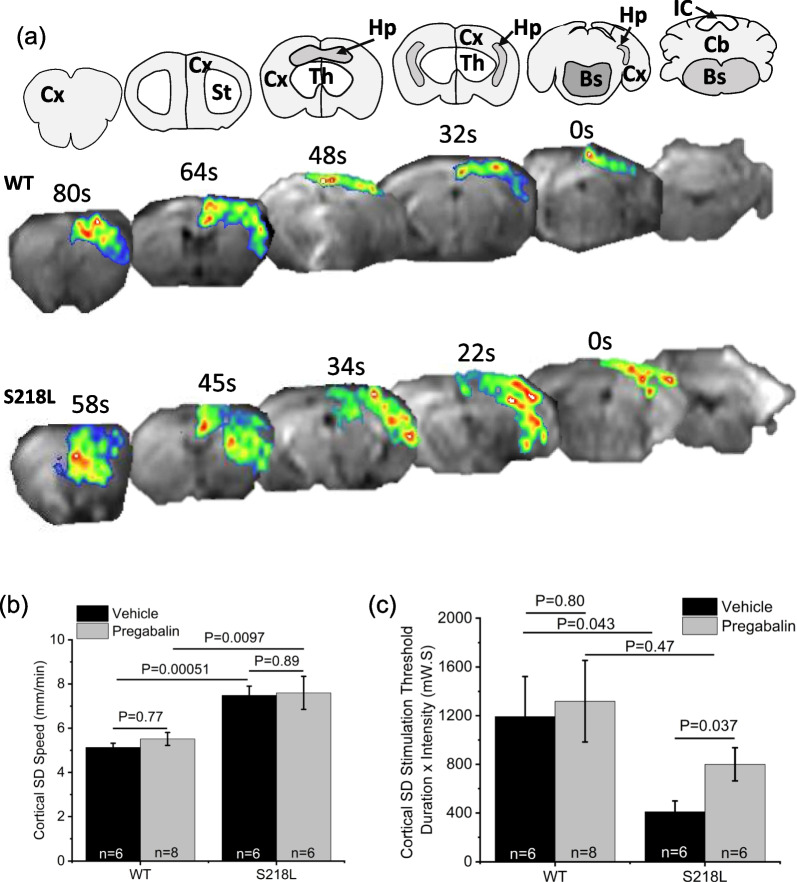
Effect of chronic pregabalin treatment on cortical SD threshold and speed. **a** Coronal maps (upper panels) corresponding to DW-MRI images (lower panels). Maps denoted for cortex (Cx), striatum (St), hippocampus (Hp), thalamus (Th), brainstem (Bs) and cerebellum (Cb) in wild-type (WT) and S218L mice. **b** Graph showing cortical SD speed. **c** Graph showing cortical SD stimulation threshold

In agreement with previous results [22, 26], in vehicle-treated mice once the threshold for cortical SD was achieved, propagation speed of SD was significantly faster in S218L compared to wild-type mice (wild-type = 5.1 ± 0.2 mm/min; S218L = 7.5 ± 0.4 mm/min; P = 0.00051**; **Fig. 2b). There was no apparent effect of chronic pregabalin treatment on cortical SD speed in either wild-type mice (5.4 ± 0.3 (n = 6); P = 0.8) or S218L mice (7.6 ± ; 0.7 (n = 5); P = 0.89).

Also in agreement with previous results, the threshold for cortical SD was significantly lower in S218L mice compared to wild-type (wild-type = 1192 ± 331 milliwatts seconds (mW.S); S218L = 410 ± 89 mW.S; P = 0.043; Fig. 2c). While there was no significant effect of pregabalin treatment on SD threshold in wild-type mice (1319 ± 336 mW.S; P = 0.80), SD threshold was significantly increased by chronic pregabalin in S218L mice (800 ± 136 mW.S; P = 0.037).

### Chronic pregabalin induces genotype-specific effects on subcortical SD propagation

**Fig. 3:**
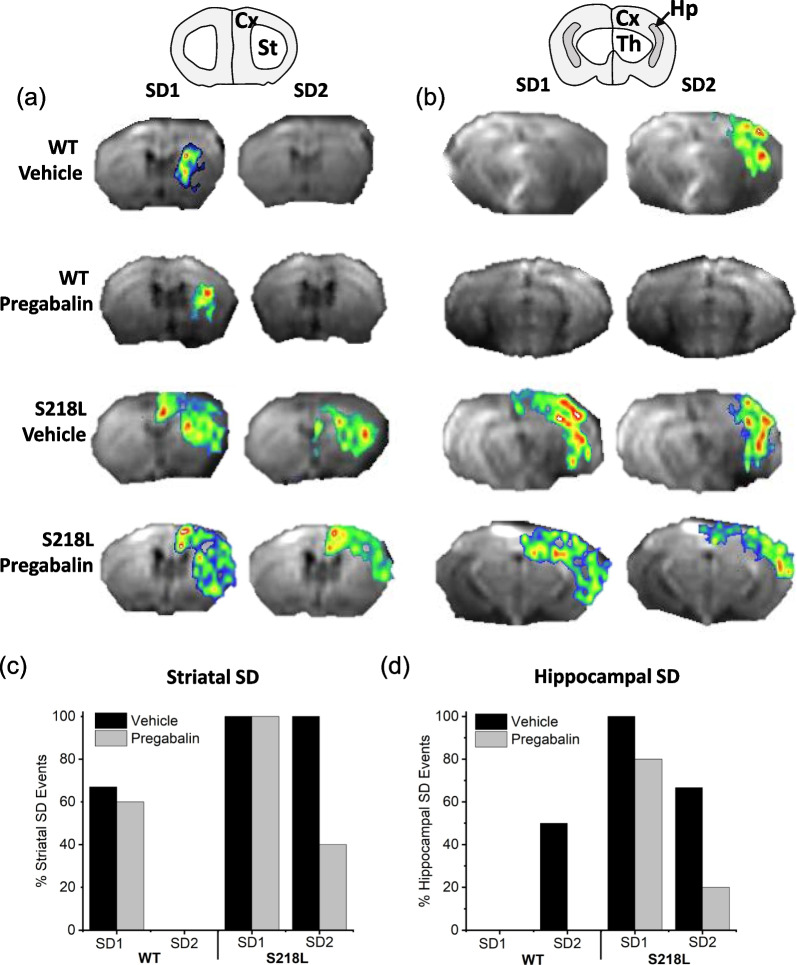
Effect of chronic pregabalin treatment on subcortical SD propagation. **a** Coronal brain map (upper panel) corresponding to DW-MRI images (lower panels) showing striatal SD propagation in wild-type (WT) and S218L mice. **b** Coronal brain map (upper panel) corresponding to DW-MRI images (lower panels) showing hippocampal SD propagation. **c** Graph summarizing striatal SD propagation events. **d** Graph summarizing hippocampal SD propagation events

We previously observed that electrically-induced single SD events were constrained to the cortex in wild-type mice but further invaded subcortical brain structures in FHM-1 mice strains [22]. Here, under a non-invasive optogenetic protocol of increasing time/intensity separated by 5 min intervals many animals displayed two distinct SD events (Table 1). We hypothesized that the 1st and 2nd SD events may display distinct properties and potentially respond to pharmacological treatment with pregabalin differently and thus were analyzed separately. Examining subcortical SD propagation in vehicle treated wild-type animals the 1st SD event propagated to the striatum 67% of the time, notably following a significant delay of 97–125 s after passing through the cortex (Fig. 3c; 4 of 6 events). In contrast, none of the vehicle-treated wild-type mice showed subcortical propagation into the striatum following the 2nd cortical SD event. Examining hippocampal SD propagation in vehicle treated wild-type animals, SD never propagated into the hippocampus (0 of 6 events) on the 1st event (Fig. 3d**; **Table 1). However, 50% (2 of 4 events) of the subsequent 2nd SD events were found to invade the hippocampus. We speculate that this may occur as a result of the 2nd SD occurring in response to higher stimulation intensity and/or from a sensitization of the hippocampus to SD propagation induced by the 1st SD event.

**Table 1:** Cortical and subcortical SD events

	Number of SD Events			
	WT		S218L	
	1st SD	2nd SD	1st SD	2nd SD
Vehicle—cortex	6	4	6	6
Vehicle—striatum	4	0	6	6
Vehicle—hippocampus	0	2	6	4
Pregabalin—cortex	5	5	5	5
Pregabalin—striatum	3	0	5	2
Pregabalin—hippocampus	0	0	4	1

Summary data for animals that displayed two SD events showing number of 1st and 2nd cortical, striatal and hippocampal SD events in wild-type (WT) and S218L mice treated with either vehicle or pregabalin

Chronic pregabalin treatment did not prevent SD propagation into the striatum in wild-type mice (Fig. 3c). However, a pregabalin-dependent effect was found for the 2nd SD in that chronic treatment fully prevented SD propagation into the hippocampus (Fig. 3d).

For vehicle treated S218L animals 100% of the 1st SD events spread subcortically into both the striatum and hippocampus (Fig. 3c, d). Upon the 2nd SD there remained 100% event spread (6 events) into the striatum and 67% (4 of 6 events) spread into the hippocampus. Chronic pregabalin treatment did not prevent 1st SD event propagation (5 events) into the striatum but notably decreased 2nd event striatal spread (40%; 2 of 5 events). Hippocampal 1st event SD spread was only slightly deceased in S218L pregabalin treated animals (80%; 4 of 5 events) but was nearly entirely attenuated for 2nd hippocampal SD events (20%; 1 of 5 events).

Overall, examining the total number of subcortical 1st and 2nd SD events across strains with and without drug treatment it is apparent that chronic pregabalin has significant attenuating effects concerning subcortical SD spread in both wild-type and S218L animals.

### Chronic pregabalin has differential effects on spontaneous synaptic excitability in FHM-1 S218L compared to wild-type mice

**Fig. 4:**
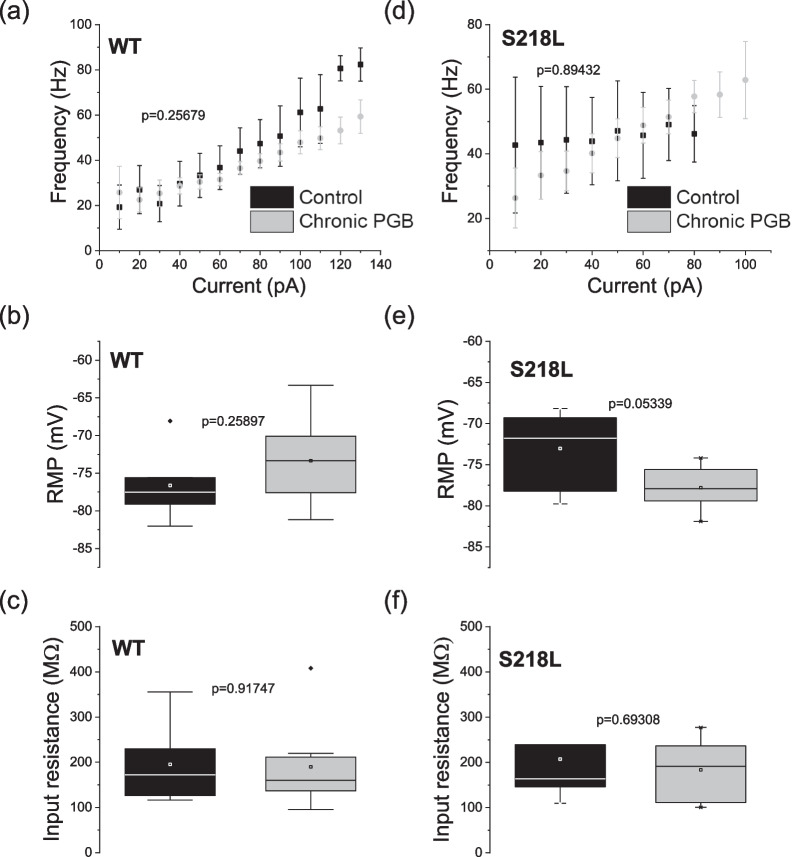
Chronic pregabalin does not affect firing frequency of CA1 hippocampal neurons of S218L FHM-1 or wild-type mice. CA1 neurons were held at their intrinsic resting membrane potential and 10 pA steps were injected starting at – 100 pA. Frequency was calculated and plotted against current injected above threshold for **a** wild-type (WT) (Control n = 6, pregabalin n = 8 neurons) and **d** S218L mice (Control n = 7 neurons, pregabalin n = 5 neurons) treated with saline (grey dots) or chronic pregabalin (black dots). Error bars indicate ± SEM. Chronic pregabalin (black) does not affect resting membrane potential (RMP) or input resistance (R_in_) of CA1 neurons from wild-type or S218L mice compared to controls (grey bars). Box plots showing **b** RMP from wild-type mice, **c** Input resistance from wild-type mice **e** RMP from S218L mice and **f** input resistance from S218L mice

To evaluate the impact of chronic pregabalin treatment on intrinsic neuronal excitability, mice were treated chronically with pregabalin (12 mg/kg/day) for 7–9 days then sacrificed for preparation of acute brain slices ex vivo. Whole-cell patch clamp electrophysiology of hippocampal CA1 neurons in the presence of picrotoxin to block inhibitory synaptic input confirmed that intrinsic membrane properties and action potential firing frequency (Fig. 4) were not significantly different between wild-type and S218L mice. Further, CA1 intrinsic properties were not significantly affected by chronic pregabalin treatment (Fig. 4a). There were no statistically significant differences in rheobase across conditions (p = 0.889, ANOVA): wild-type control: 47 ± 16 pA, S218L control 42 ± 12 pA, wild-type pregabalin 41 ± 12 pA, S218L pregabalin 56 ± 17 pA. Representative recordings are shown in Additional file 1: Fig. S1.

**Fig. 5:**
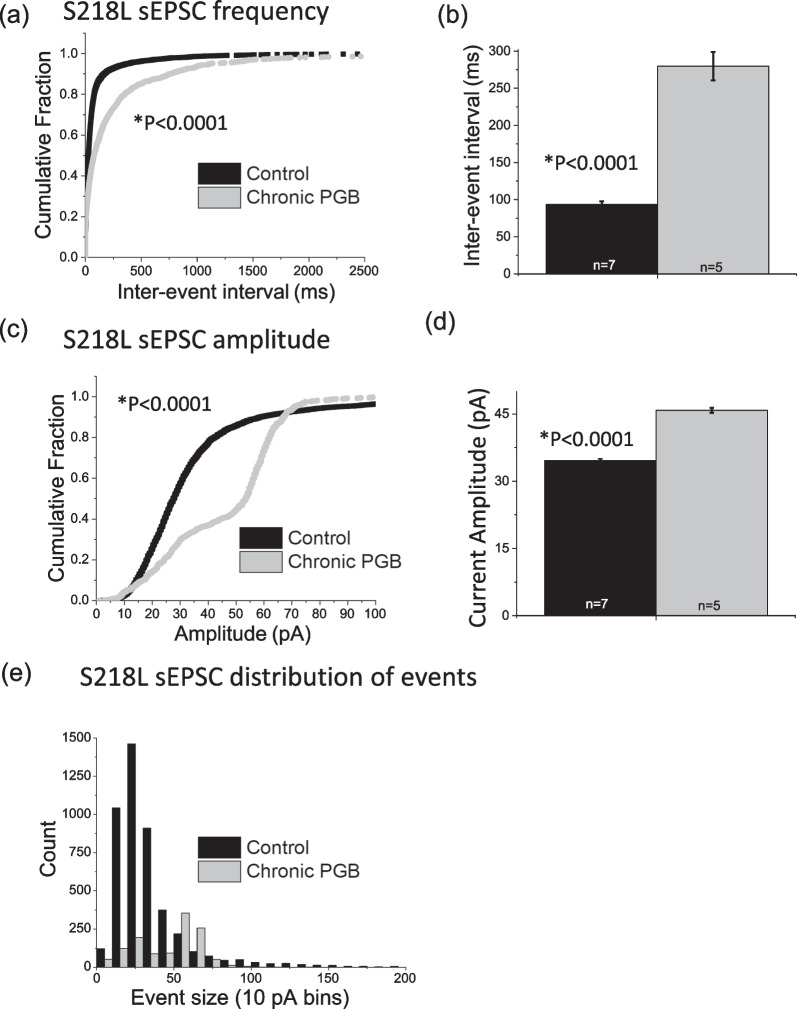
Effect of chronic pregabalin treatment on CA1 hippocampal spontaneous excitatory postsynaptic currents recorded from S218L FHM-1 mice. **a**, **b** Chronic pregabalin (PGB) significantly decreases frequency (increases Inter-event Interval, p < 0.0001) of sEPSCs in S218L CA1 neurons. This was confirmed by analyzing both **a** cumulative probability plots (K-S test) and **b** mean values (t-test). **c**, **d**, **e** In S218L mice, chronic pregabalin decreases amplitude of smaller events (< 50 pA) and increases amplitude of larger (50–70 pA) events. This was confirmed by analyzing **c** cumulative probability plots (K-S test), **d** mean values (t-test) and **e** sEPSC event distribution. Error bars indicate ± SEM. Control = black, chronic pregabalin = grey

**Fig. 6:**
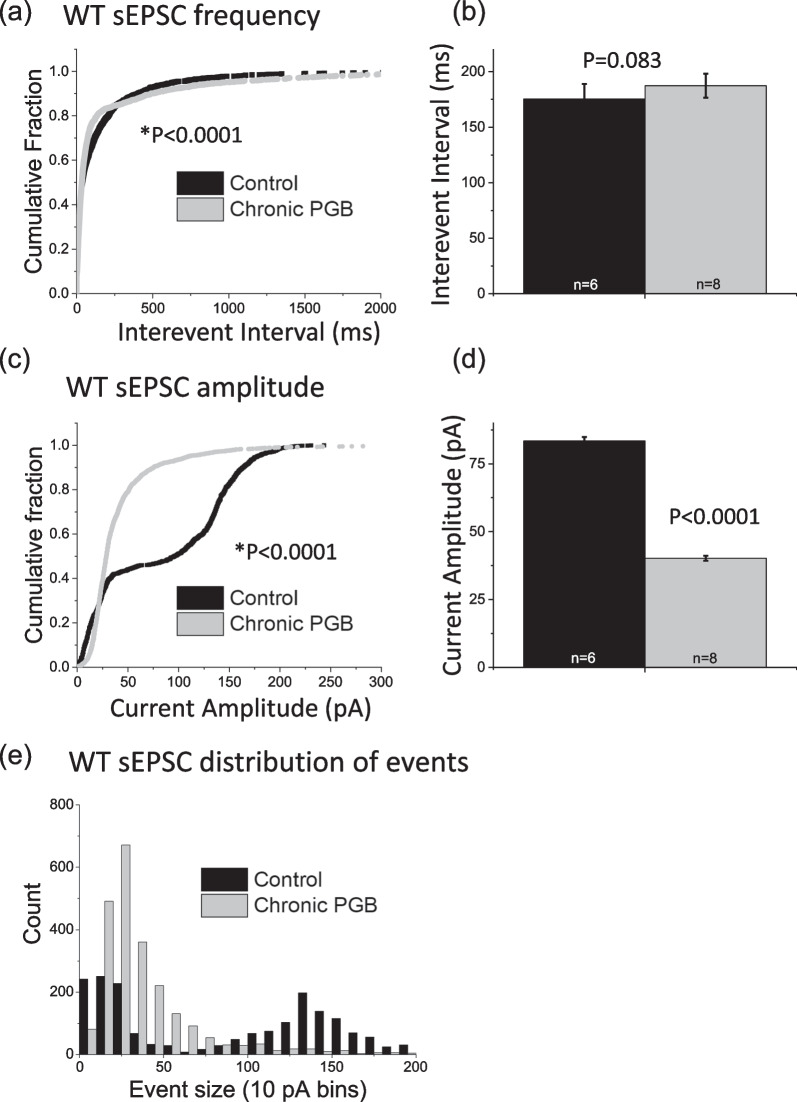
Effect of chronic pregabalin treatment on CA1 hippocampal spontaneous excitatory postsynaptic currents recorded from wild-type mice. **a**, **b** Chronic pregabalin (PGB) decreases frequency (increases Inter-event Interval p < 0.0001) when comparing** a** cumulative distributions (K-S test), but has no significant effect on **b** mean values (t-test). **c**, **d** Chronic pregabalin significantly decreases amplitude of sEPSCs in wild-type (WT) CA1 neurons. This was confirmed by analyzing both **c** cumulative probability plots (K-S test), **d** mean values (t-test) and **e** sEPSC event distribution. In wild-type-mice, chronic pregabalin increases amplitude of events (20–80 pA) and decreases amplitude of < 10 pA events and > 80 pA events. Error bars indicate ± SEM. Control = black, chronic pregabalin = grey

Examining glutamatergic synaptic excitability, as expected [22] sEPSC frequency (i.e. inverse of inter-event interval) was significantly higher in S218L CA1 neurons compared to wild-type neurons (wild-type = 160 ± 11 ms; S218L = 93 ± 4.5 ms; P < 0.0001). Chronic pregabalin treatment significantly reduced sEPSC frequency in S218L CA1 neurons (pregabalin = 280 ± 19 ms; vehicle control = 93 ± 4.5 ms, P < 0.0001, Fig. 5a, b), but had no effect on the sEPSC frequency in wild-type neurons (pregabalin = 187 ± 11 ms; vehicle control = 175 ± 14 ms, P = 0.08, Fig. 6a, b).

sEPSC amplitude was significantly smaller in S218L CA1 neurons compared to wild-type neurons (wild-type = 83 ± 1.5 pA; S218L = 35 ± 0.4 pA; P < 0.0001). Interestingly, chronic pregabalin reduced sEPSC amplitude in wild-type CA1 neurons (wild-type control = 83 ± 1.5 pA and wild-type + pregabalin = 40 ± 0.9 pA, P < 0.0001; Fig. 6c, d), whereas in CA1 neurons from S218L mice sEPSC amplitude was significantly increased by chronic pregabalin treatment (S218L control = 35 ± 0.4 pA and S218L pregabalin = 46 ± 0.6 pA; P < 0.0001**; **Fig. 5c, d**).**

Further analysis of the distribution of sEPSC event sizes revealed that chronic pregabalin increased the number of small events (< ~ 100 pA, Fig. 6e) in wild-type CA1 neurons, but decreased the number of smaller events in S218L neurons (< ~ 50 pA, Fig. 5e).

### Plasma levels with acute versus chronic pregabalin

In order to compare systemic exposure of acute versus chronic pregabalin treatment, we measured plasma pregabalin in wild-type mice that had received a single acute dose of pregabalin (160 mg/kg, i.p.) and those treated with subcutaneous chronic pregabalin (12 mg/kg/day, 7–9 days). Acute pregabalin treatment resulted in plasma concentrations of 544.9 ± 96.5 µM (n = 5), whereas chronic pregabalin treatment resulted in plasma concentrations of 1.2 ± 0.2 µM (n = 4; P = 0.001). These results show that the serum concentration of pregabalin at the shorter time used for acute, high-dose pregabalin treatment was far higher than that obtained with chronic treatment at the more clinically relevant dose used here.

## Discussion

In the current study, the effects of chronic pregabalin (12 mg/kg/day for 7–9 days) versus vehicle were examined in wild-type control and S218L FHM-1 knock in mice employing optogenetic stimulation and DW-MRI to track various parameters associated with SD across the entire brain in vivo. In order to ensure SD initiation, multiple optogenetic pulses of increasing time and power separated by 5-min intervals were delivered in each animal (see Methods). Notably, the optogenetic protocol often resulted in two distinct cortical SD events (labelled 1st and 2nd; summarized in Table 1) and that were analyzed separately concerning subcortical spreading and chronic pregabalin effects.

Assessing the 1st SD event and comparing vehicle treated wild-type and FHM-1 strains using optogenetic stimulation we confirmed both a lower threshold for SD initiation and faster SD speed in S218L knock-in mice compared to wild-type (Fig. 2; Table 2). These results reflect and support previous studies indicating gain-of-function pathophysiological effects of the S218L mutation on P/Q-type calcium channels including increased calcium conductance at resting membrane potentials together with constitutive calcium-dependent facilitation at synaptic terminals [27–30].

**Table 2:** Effects of chronic pregabalin on SD

	Effect of Chronic Lower-Dose Pregabalin vs Vehicle (Opto-Stim)			
	WT		S218L	
	1st SD	2nd SD	1st SD	2nd SD
Cortical SD Threshold	↔	N/A	↑	N/A
Cortical SD Speed	↔	N/A	↔	N/A
Striatal SD Invasion	↔	↓	↔	↓
Hippocampal SD Invasion	X	↓	↓	↓

Summary of effects of acute pregabalin (data taken from Cain et al. PNAS [22]) vs chronic pregabalin (data taken from current study). X = no SD in vehicle-treated animals. ↑ = increase with pregabalin, ↓ = decrease with pregabalin, ↔  = no effect of pregabalin. N/A = not applicable as cortical speed and threshold were only measured for 1st SD events

Compared to vehicle, chronic pregabalin treatment did not affect cortical SD speed in wild-type mice (Fig. 2). This agrees with our previous acute dosing pregabalin study showing no effect on cortical SD speed [22] and together suggest that for wild-type animals pregabalin across a range of acute and chronic dosing levels does not affect the underlying mechanisms driving cortical SD speed. In contrast to the previous study that found acute gabapentin increased the threshold for SD in wild-type mice [22], here under chronic dosing there was no effect on cortical SD threshold. Factors to consider given the differences across studies and pregabalin-mediated effects in wild-type mice include that: (1) the mechanisms underlying cortical SD threshold are likely distinct from those driving cortical SD speed, (2) the dosing regimen and plasma levels of pregabalin critically affect the initiation of SD events, and (3) the pregabalin concentration obtained by chronic administration at 12 mg/kg/day (~ 1.2 μM) may have been insufficient to fully engage P/Q-type α2δ-1 subunits to mechanistically affect the higher SD threshold in wild-type mice.

Like that for wild-type mice, chronic pregabalin treatment in S218L mutant mice had no effect on cortical SD speed (Fig. 2). In contrast to wild-type, chronic pregabalin significantly increased cortical SD threshold (Fig. 2). These results are distinct from the previous acute dosing study where pregabalin showed no effect on cortical SD threshold but significantly decreased SD speed. Combined, these data further support the notion that mechanisms underlying cortical speed and threshold are distinct and indicate that even the lower pregabalin levels obtained by chronic dosing are sufficient to attenuate cortical SD initiation.

While the findings indicate treatment-specific (acute high dose vs chronic lower dose) and genotype-specific differences in SD threshold and cortical SD speed, further study is required to determine the underlying reasons. In part, the differential effects may relate to the fact that gabapentinoids can both bind to HVA calcium channel α2δ-1subunits to acutely block calcium channel activity and under chronic dosing conditions to further downregulate HVA channel trafficking to the plasma membrane [7, 22, 31].

Examining subcortical spread and considering the 1st and 2nd SD events showed that for wild-type vehicle-treated mice most animals exhibited 1st SD event invasion into the striatum but never for the 2nd SD event (Fig. 3). Contrastingly, in these animals the 2nd SD event never invaded the striatum but in some animals was found to spread into the hippocampus. Interestingly, chronic pregabalin treatment did not affect the 1st SD event regarding striatal SD spread but entirely attenuated hippocampal invasion for the 2nd SD event (Fig. 3, Table 2).

That striatal SD was observed during the 1st but not 2nd SD event in wild-type animals suggests that in the nonpathological brain this region is basally more resistant to SD invasion and/or rapidly develops a resistance to subsequent SD events following the initial subcortical SD propagation event. Hippocampal SD was observed in 50% of wild-type animals on the 2nd SD event (in response to a higher stimulation intensity) but not the first SD event, suggesting that a higher stimulation intensity may be required to initiate a second SD event in this region. Alternatively, it is possible that the hippocampus of wild-type animals rapidly becomes sensitized to subsequent SD events. Indeed, it has been shown that multiple SDs can increase phosphorylation of Src family kinases (SFKs) to drive TRPA1 signaling and sensitize trigeminovascular pathways in migraine [32, 33].

Examining subcortical SD spread in S218L animals following optogenetic stimulation showed that under vehicle-treated conditions both the 1st and 2nd SD events invaded the striatum in 100% of animals (Fig. 3). The 1st SD event also spread into the hippocampus in 100% of S218L mice with the 2nd SD event invading the hippocampus in most animals (4 of 6). Chronic pregabalin did not affect 1st SD event spread into the striatum but did attenuate 2nd SD spread in 3 of 5 animals. Chronic pregabalin similarly affected 2nd SD spread in the hippocampus, having little effect on the 1st SD event invasion but largely attenuating 2nd SD spread into this region (Fig. 3 and Tables 1, 2). The attenuating effects of chronic pregabalin treatment on 2nd SD subcortical spread in S218L mice contrasts with our previous study using electrical stimulation where acute high dose pregabalin had no affect in S218L mice but completely prevented striatal and hippocampal subcortical spread in the milder R192Q strain [22]. Recently it has been shown that optogenetically-evoked SD initiates nuclear release of the neuroinflammatory mediator HMGB1 in S218L mice [34]. It is possible that repeated SD events evokes the release of more HMGB1 and that since pregabalin can inhibit cytoplasmic translocation of HMGB1 that pregabalin is more effective following repeated SDs [35, 36].

Cortical SD events have been proposed as a protective mechanism to limit hyperexcitability during for example seizures [37] and repeated cortical SD events can reduce the susceptibility to subsequent SD events [33]. On the other hand, we recently demonstrated that SD propagation from the superior colliculus to the brainstem correlates with respiratory arrest and death in S218L mice [25] and that this strain is sensitive to seizure-induced fatality [17]. As such, future studies should explore whether pregabalin can prevent SD propagation into the brainstem, and provide a potential preventative treatment for Sudden Unexpected Death in Epilepsy.

Of relevance to the distinct pathophysiologies associated with specific FHM-1 mutations [38], a considerable delay was observed between SD spread from the cortex into the adjacent striatum in wild-type mice but not in S218L mice. A similar time delay between cortical and striatal SD invasion was observed with mice harboring the milder FHM-1 R192Q mutation [22], suggesting that R192Q and wild-type brains may share similar mechanisms governing subcortical SD propagation.

Chronic pregabalin treatment resulted in a decrease in sEPSC frequency in S218L mice ex vivo hippocampal brain slices. This agrees with previous observations where acute pregabalin (100 µM) applied to treatment-naïve brain slices decreased sEPSC frequency in FHM-1 R192Q and S218L CA1 neurons but had no effect on wild-type neurons. While the inhibitory effect on sEPSC frequency in S218L CA1 neurons is consistent with the concomitant slowing of cortical SD speed in acute pregabalin-treated animals, a similar correlation was not observed with chronic pregabalin treatment. This may indicate either that the effects observed on hippocampal synaptic excitability are differentially affected by acute effects on channel conductance vs chronic effects on trafficking/internalization or that these effects are not necessarily related to cortical SD speed. We observed that chronic pregabalin treatment increased sEPSC amplitude in S218L CA1 neurons but not in wild-type. This is consistent with the effect of acute pregabalin and we speculate may be related to homeostatic synaptic scaling whereby the chronic blockade of synaptic activity produces a compensatory increase in sEPSC amplitude [39]. It has been shown that α2δ-1 subunits are critically involved in regulating homeostatic plasticity in cultured hippocampal neurons [31]. Pilch et al., demonstrated that during homeostatic synaptic plasticity endogenous levels of α2δ-1 decreased and conversely, α2δ-1 overexpression prevented homeostatic synaptic plasticity in hippocampal neurons. That this effect is specific to S218L mice and not observed under wild-type conditions perhaps indicates that pathophysiologically the S218L mutation alters α2δ-1 subunit expression in hippocampal neurons. In numerous rodent pain models α2δ-1 subunit expression is increased as a result of the introduced insult [40–42]. While the therapeutic relevance of the effects of pregabalin on synaptic activity in CA1 neurons and with respect to SD remains to be fully elucidated, the current data nevertheless provide insight into the pathophysiology of FHM-1 models.

In summary, the present study combined DW-MRI and optogenetics finding distinct SD patterns of subcortical propagation upon initial and subsequent SD events. Further, that the subsequent events can be suppressed by chronic pregabalin treatment is more consistent with clinical doses where plasma concentrations can be as low as 1 µg/ml (~ 6 µM) pregabalin [43]. Chronic pregabalin treatment resulted in genotype-specific effects on spontaneous glutamatergic synaptic excitability at CA1 hippocampal synapses. These findings provide further insight into the pathophysiological mechanisms underlying SD and raise the potential that therapeutic approaches to limit SD propagation might be effective in indications such as FHM-1, migraine, epilepsy and traumatic brain injury. That the brain levels of pregabalin achieved here with chronic dosing were lower than for our previous acute dosing study likely accounts for some of the differences between SD parameters and subcortical spread between studies. Together they emphasize the importance of performing drug treatment studies as therapeutically relevant as possible.

## Methods

### Animals

All experiments were performed in accordance with the guidelines of the Canadian Council on Animal Care and the University of British Columbia Animal Care Committee. Heterozygous breeding of transgenic Cacna1a^Wild−type/S218L^ mice produced male and female, wild-type and Cacna1a^S218L/S218L^ littermates for use in all experiments at postnatal day (P) 25–P40 mice and were genotyped as previously described [16].

### Surgery

Animals were anaesthetized using dexmedetomidine/midazolam/fentanyl anesthesia and placed in a stereotaxic frame and an incision made in the scalp. A hole was drilled in the skull over the right hemisphere, over the visual cortex and 0.5 µl AAV5-hsyn-hChR2(H134R)-EYFP [AAV-ChR2; Canadian Neurophotonics Platform Viral Vector Core Facility (RRID:SCR_016477)] injected unilaterally into the visual cortex (in mm) = bregma−3.0, midline 1.5, depth (from brain surface) 0.5. The scalp incision was sutured and the animal monitored for a two-week recovery period.

### Magnetic resonance imaging

Two-weeks following AAV-ChR2 injection, animals were anesthetized using isoflurane and an incision was made in the scalp. For stimulation of the visual cortex a port (Shanghai Laser & Optics Century) was glued onto the skull, 0.3 mm posterior to the hole over the injected area to accommodate a fiberoptic ferrule, taking care to leave a cement-free area for the laser path to the skull. To transfer onto an injectable anesthetic, animals were then injected with urethane (30%, 8 µl/g mouse weight) and isoflurane gradually decreased to 0% over 2 min. A minimum period of 20 min was applied between cessation of isoflurane and initiation of scanning. During scanning animals breathed unaided on normal air delivered to the scanner by facemask.

Diffusion-Weighted Magnetic Resonance Imaging (DW-MRI) was undertaken using a 7 Tesla animal scanner (Bruker Biospin Ltd.). A quadrature radiofrequency coil with 70-mm inner diameter volume was used for pulse transmission and the MRI signal was received with a 14-mm diameter actively decoupled surface coil. The mouse was laid supine in the MRI cradle with the fiberoptic cable fed through the center of the radiofrequency coil and connected to the port or cannula ferrule. Respiratory rate, heart rate and body temperature were monitored during scanning with a Model 1025 Control/Gating system (SA Instruments). DW-MRI was acquired using DW spin-echo planar imaging (EPI) with a b-value of 1800s/mm^2^ (echo time/repetition time = 29/2000 ms, two shots, field of view = 2 × 2 cm, matrix size = 64 × 64, slice thickness = 1.25 mm, eight interleaved slices). Two shots resulted in a 4-s time resolution.

Upon initiation of scanning, a 1-min baseline was acquired followed by stimulation for 2 s at 10 mW; measured on four different fiberoptic cables utilized throughout study. Stimulation intensity was first applied for 2 s at 40 mW and subsequent increasing stimuli applied in 5-min intervals as follows: 2 s at 80 mW 2 s at 110 mW, 5 s at 110 mW, 10 s at 110 mW until the stimulation threshold for Spreading Depolarization (SD) was reached. Since it is not possible to determine whether SD has occurred until after the scanning protocol had completed and the MRI data reconstructed, all stimulations were applied in every animal before evaluating SD occurrence. Following the initial SD a second cortical SD was observed in some but not all instances. Cortical speed and threshold measurements were calculated for the 1st SD event in all cases. For subcortical spread the 1st and 2nd SD events were analyzed separately.

### Acute brain slice preparation

Slices were prepared as previously described [22]. Briefly, brains were removed from rats following death by anesthesia using isoflurane [5% in oxygen (vol/vol)] and cervical dislocation. The brain was then immediately transferred to ice-cold sucrose-artificial cerebral spinal fluid (sucrose-aCSF) containing 214 mM sucrose, 26 mM NaHCO_3_, 1.25 mM NaH_2_PO_4_, 11 mM glucose, 2.5 mM KCl, 0.5 mM CaCl_2_, 6 mM MgCl_2_, bubbled with 95% O_2_:5% CO_2_. Brain tissue was glued to a cutting chamber in a vibrating microtome (VT 1200; Leica), which then was filled with ice-cold sucrose-aCSF.

### Acute brain slice electrophysiology

Slice whole-cell patch-clamp electrophysiology recordings were performed as previously described [22]. Briefly, horizontal slices (350 μm thick) were cut from the ventral hippocampus, incubated at 33–35 °C in aCSF bubbled with 95% O_2_:5% CO_2_, and for experiments transferred to a recording chamber superfused with aCSF and maintained at 33–35 °C. CA1 neurons were identified by infrared-differential intereference contrast (IR-DIC) optics (SliceScope 6000; Scientifica). For recording a Multiclamp 700B amplifier (Molecular Devices) was used. Pipette resistance was 4–6 MΩ. Internal solution contained (in mM): 120 K-gluconate, 10 Hepes, 1 MgCl_2_, 1 CaCl_2_, 11 KCl, 11 EGTA, 4 MgATP, and 0.5 NaGTP, pH 7.2, osmolarity 290 mOsm. Membrane potential responses under current-clamp conditions were sampled at 50 kHz and filtered at 10 kHz and bridge balance values > 20 MΩ were discarded. Under voltage clamp conditions, data acquisition was sampled at 20 kHz and filtered at 2.4 kHz. Recordings with a series resistance greater than 20 MΩ were discarded.Spontaneous excitatory postsynaptic currents (sEPSCs) were measured from CA1 hippocampal neurons of WT and S218L mice as per [22]. To evaluate sEPSCs, cells were held at a membrane potential of − 70 mV. Data acquisition was sampled at 20 kHz and filtered at 2.4 kHz. Recordings with a series resistance greater than 20 MΩ were discarded, and series resistance was compensated to 70%. Electrophysiological data analysis was performed using Clampfit (v9 and v10; Molecular Devices). Cumulative distributions were compared using the Kolmogorov–Smirnov test. Data are plotted as mean ± standard error. The recording duration for sEPSCs was 1 min. For constructing the histogram sEPSCs were sorted into 10 pA bins.

### Chronic pregabalin treatment

Pregabalin or vehicle (saline) was administered by osmotic minipump (Alzet, Model 1007D) planted subcutaneously at 0.5 µl/Hr for 7–9 days at a daily dose of 12 mg/kg. The minipump was implanted under isoflurane anesthesia 7–9 days prior to the DW-MRI imaging procedure.

### Measurement of serum pregabalin levels

A modification of the procedure described by Alles et al. [35] for analysis of gabapentin was employed. Standards were prepared in drug-naïve serum. Pregabalin-treated samples and standards (consisting of varying amounts of pregabalin added to drug-free serum) were brought up to 100 µl with Millipore-filtered water. An equal volume of MeOH/acetonitrile/formic acid (1:1:0.04) was added and the samples and standards were vortexed well and left on ice for 10 min. After centrifugation at 15000xg for 5 min, supernatants were each transferred to an HPLC vial and 10 µl injected. Analysis was performed using a Waters ZQ Mass detector fitted with an ESCI Multi-Mode ionization source and coupled to a Waters 2695 Separations module (Waters, Milford, MA, USA). HPLC separation was performed on an Atlantis T3 (3 μm, 3.0 × 100 mm) column (Waters, Milford, MA, USA) with a guard column of similar material. Mobile phase A consisted of 0.05% formic acid in water and mobile phase B was composed of 0.05% formic acid in acetonitrile. Initial conditions were 85% A and 15% B at a flow rate of 0.3 ml/min. A gradient was run increasing to 100% B in 8 min followed by a return to initial conditions. Electrospray parameters: Capillary voltage 3.25 kV; Rf lens voltage 1.0 V; source 110 ℃; desolvation temperature 350 ℃; cone gas flow (nitrogen)100 l/Hr; desolvation gas flow (nitrogen) 400 l/Hr; cone voltage, 9.

### Ex vivo imaging confirmation of channelrhodopsin expression

At the end of the experiment mice were euthanized with urethane, transcardial perfusion performed with paraformaldehyde (4%), the brain removed and transferred to paraformaldehyde (4%) for 24 h before being transferred to PBS for a minimum of 2 days. Brains were then cut into slices (300 µm thickness) using a vibrating blade microtome (VT-1200S, Leica) in the sagittal plane. Imaging was performed using a Zeiss AxioZoomV16 fluorescence stereo microscope with a Plan-NEOFLUAR Z 2.3x/1.5 NA objective using a GFP/YFP filter set (FS38: excitation 470/40, emission 525/50).

### Data and statistical analysis

Electrophysiological data analysis was performed using Clampfit (v10, Molecular Devices). DW-MRI analyses were performed using MATLAB (v 2014a, Mathworks) and and ImageJ (v 1.50d, NIH). Graphing and statistical analyses were performed using Origin (v8.6, OriginLab). Data followed a normal distribution and statistical significance was calculated using Student’s two-sample t-test (paired where relevant). One-Way ANOVA with Tukey’s post-hoc test was used for multiple comparisons. Data are plotted as mean ± standard error.

### Supplementary Information

**Additional file 1: Figure S1.** Representative whole-cell patch-clamp recordings from CA1 neurons in acute brain slices. Left. Representative current clamp recording showing response to 10 pA stepwise current injection. Right. Representative voltage clamp recordings of spontaneous excitatory postsynaptic currents (sEPSCs).

## Data Availability

The datasets used and/or analysed during the current study are available from the corresponding author on reasonable request.

## Abbreviations

SD: Spreading depolarization
HVA: High voltage-activated
FHM-1: Familial hemiplegic migraine type 1
