# Supplementary material for: Chronic pregabalin treatment protects against spreading depolarization and alters hippocampal synaptic characteristics in a model of familial hemiplegic migraine-type 1
Source: Mol Brain. 2023 Nov 3;16:76. doi: 10.1186/s13041-023-01062-6 (PMC10623724; doi:10.1186/s13041-023-01062-6)
Supplement: Supplementary file 1 — Additional file 1: Figure S1. Representative whole-cell patch-clamp recordings from CA1 neurons in acute brain slices. Left. Representative current clamp recording showing response to 10 pA stepwise current injection. Right. Representative voltage clamp recordings of spontaneous excitatory postsynaptic currents (sEPSCs). [file 13041_2023_1062_MOESM1_ESM.pdf]

Figure S1

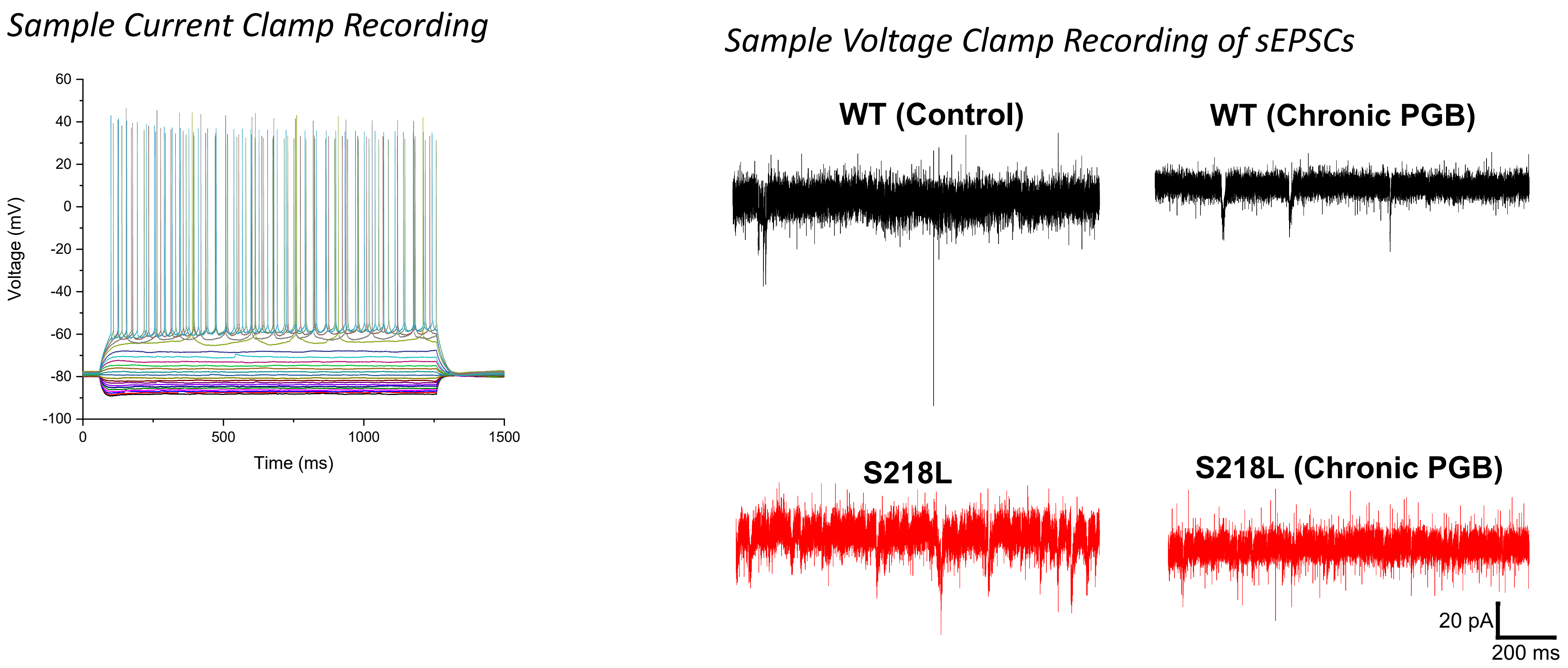

Figure S1. Representative whole-cell patch-clamp recordings from CA1 neurons in acute brain slices. **Left.** Representative current clamp recording showing response to 10 pA stepwise current injection. **Right.** Representative voltage clamp recordings of spontaneous excitatory postsynaptic currents (sEPSCs).
